# Retraction: Novel Orthobunyavirus Causing Severe Kidney Disease in Broiler Chickens, Malaysia, 2014–2017

**DOI:** 10.3201/eid2608.202331

**Published:** 2020-08

**Authors:** Vilmos Palya, Edit Walkóné Kovács, Szilvia Marton, Tímea Tatár-Kis, Balázs Felföldi, Barbara Forró, Marianna Domán, Krisztián Bányai

**Affiliations:** Ceva-Phylaxia Veterinary Biologicals Co. Ltd., Budapest, Hungary (V. Palya, E.W. Kovács, T. Tatár-Kis, B. Felföldi);; Hungarian Academy of Sciences, Budapest (S. Marton, B. Forró, M. Domán, K. Bányai)

**Keywords:** Peribunyaviridae, Orthobunyavirus, Turlock serogroup, Kedah fatal kidney syndrome virus, broiler, chickens, viruses, Malaysia

**To the Editor:** We would like to retract our article Novel Orthobunyavirus Causing Severe Kidney Disease in Broiler Chickens, Malaysia, 2014–2017 from the June 2019 issue of Emerging Infectious Diseases (*1*). We assert that research was performed in good faith, that all the experimental data contained in the article are well founded and scientifically valid, and that there was no scientific misconduct. However, we subsequently were made aware of further information about the epidemiologic and clinical observations made locally in Malaysia, which brings into question the geographic location where the noted virus originated. Thus, we request this article’s retraction.
